# The Editor's Letter-Box

**Published:** 1919-10-04

**Authors:** 


*To the Editor of* THE HOSPITAL.

Queen Charlotte's Hospital,

Marylebone Road, N.W.

*September* 23, 1919.

---

*To the Editor of* THE HOSPITAL.

SIR,—With regard to the criticism in THE HOSPITAL *re* the nursing hours, cleanliness, and defective food supply at Queen Charlotte's Hospital, might I voice my opinion *re* conditions within the last ten months. I worked there for a period of six months as pupil-midwife. Previous to going to Queen Charlotte's I worked in a London hospital, and at the present time I am working in a London hospital. I have found the food in the three hospitals to be much the same—plentiful, plain, and wholesome. As regards the off-duty time, I never missed having two hours off duty every day, also time off to attend lectures, and one whole day every ten days. As regards the cleaning, there is very little to do in comparison to the cleaning one has to do in a general hospital. I never found any vermin during my stay in the hospital, nor did I hear of any nurse that did during my time, and all I can say is that the whole story is a tissue of lies. There were times when I felt unhappy, simply because I resented the discipline; but now, when I look back, I can see how very necessary it was, and I am more than pleased that I did my training at Queen Charlotte's. They complain of the payment of £40, they knew what the fee was before they entered, also that each pupil-midwife pays for personal laundry. When I made application for training I had a form of rules and regulations sent to me explaining all this; if the fee was too high there are other hospitals. Why did they enter Queen Charlotte's? I paid, and I consider it was money well spent. Nothing was allowed in the hospital that would be detrimental to the patients; it is as it should be, the patients come first. I consider the work done at Queen Charlotte's to be worthy of all praise and consideration. The matron at Queen Charlotte's is very kind, and also very just, and she has the interest of each pupil at heart; a better matron I never wish to work for.—Yours truly, NURSE FOWLER.

